# Systematic Analysis of Intestinal MicroRNAs Expression in HCC: Identification of Suitable Reference Genes in Fecal Samples

**DOI:** 10.3389/fgene.2019.00687

**Published:** 2019-08-13

**Authors:** Hui Wang, Yuan Lv, Cao Wang, Dongjing Leng, Yan Yan, Moyondafoluwa Blessing Fasae, Syeda Madiha Zahra, Yanan Jiang, Zhiguo Wang, Baofeng Yang, Yunlong Bai

**Affiliations:** ^1^Department of Pharmacology (State-Province Key Laboratories of Biomedicine–Pharmaceutics of China, Key Laboratory of Cardiovascular Medicine Research, Ministry of Education), College of Pharmacy, Harbin Medical University, Harbin, China; ^2^Chronic Disease Research Institute, Translational Medicine Research and Cooperation Center of Northern China, Heilongjiang Academy of Medical Sciences, Harbin, China

**Keywords:** intestinal microRNAs, quantitative real-time PCR assays, reference genes, hepatocellular carcinoma, feces

## Abstract

Hepatocellular carcinoma (HCC) is an extremely fatal malignancy. Intestinal microRNAs, which can be detected in fecal samples in humans may be involved in the pathological process of HCC. Therefore, screening for functional intestinal microRNAs in fecal samples and investigating their potential roles in the molecular progression of HCC are necessary. Quantitative real-time PCR (qRT-PCR) has been widely used in microRNA expression studies. However, few genes have been reported as reference genes for intestinal microRNAs in fecal samples. In order to obtain a more accurately analyzed intestinal microRNAs expression, we first searched for reliable reference genes for intestinal microRNAs expression normalization during qRT-PCR, using three software packages (GeNorm, NormFinder, and Bestkeeper). Next we screened and predicted the target genes of the differentially intestinal microRNAs of control and HCC mice through quantitative RT-PCR or miRtarBase. Finally, we also analyzed the mRNA targets for enrichment of Gene Ontology (GO) terms and Kyoto Encyclopedia of Genes and Genomes (KEGG) pathways using the DAVID Bioinformatic Resources database. This study has successfully screened relatively suitable reference genes and we have discovered that the differential intestinal microRNAs play significant roles in the development of HCC. The top reference genes identified in this study could provide a theoretical foundation for the reasonable selection of a suitable reference gene. Furthermore, the detection of intestinal microRNAs expression may serve as a promising therapeutic target for the diagnosis and treatment of HCC.

## Introduction

Hepatocellular carcinoma (HCC) is one of the most common cancers in the world, and the overall 5-year survival rate for HCC is less than 12% (Torre et al., 2015). It is usually diagnosed at the late stage and is characterized by a low resection rate, high postoperative recurrence, and poor drug treatment results (Galun et al., 2015). At present, the only drug approved for the treatment of liver cancer is sorafenib. Oxaliplatin has been shown to be effective in the treatment of advanced liver cancer, but the drug resistance potentially limits its efficacy (Petrelli et al., 2014). Finding sensitive and specific early diagnostic biomarkers, and understanding the mechanisms involved in the development and progression of HCC are a major issue in need of urgent resolution.

MicroRNAs are small non-coding RNAs, with transcripts that are just 18-25 nucleotides in length, and they bind mainly to the 3′ untranslated regions (3′ UTRs) of target RNAs through the microRNAs’ seed sequences (microRNA nucleotides 2–7) (Parodi et al., 2016). They are highly conservative and tissue-specific and exists widely in nematodes, drosophila, and plants, as well as in humans (Gillan et al., 2017; You et al., 2018). MicroRNAs regulate the expression of protein-coding gene expression in a sequence-specific manner through cleavage or translational repression in the genomes (Kagiya, 2016; Xiao et al., 2018). The abnormal expression of microRNAs is related to many diseases, including cancer, and miRNAs can also be used as tumor-suppressor genes or oncogenes. During the development and progression of human cancers, microRNAs have been observed to regulate cell proliferation, survival, differentiation, invasion and metastasis (Davis-Dusenbery and Hata, 2010). In patients with HCC caused by a hepatitis B virus infection, mir-29c could control cell proliferation and leads to apoptosis by targeting TNFAIP3 (, Wang et al., 2011); mir-101 produces a pro-apoptotic effect by targeting Mcl-1 (Su et al., 2009). Since the discovery of their essential roles in the occurrence and development of diseases, microRNAs have been intensely studied as prognostic and diagnostic biomarkers and predictors of drug responses (Li et al., 2014).

Past research has focused mainly on post-transcriptional and translational controls regulated by non-coding RNAs (Ranjha and Paul, 2013). MicroRNAs could efficiently interrupt the synthesis of proteins excluding further transcriptional activation and any subsequent mRNA processing steps. Therefore, it provides cells with a more accurate, direct and energy-efficient way to manipulate protein expression (Xi et al., 2007; Ge et al., 2015; Connerty et al., 2016). Liu et al. (2016) have illustrated that microRNAs in feces, which originate from intestinal cells, can enter into bacteria and regulate their gene expression and growth. These special microRNAs are called intestinal microRNAs. They specifically show that mir-515-5p and mir-1226-5p can promote the growth of *Fn* and *E. coli*, respectively. Thus, knowing the differential expressions of intestinal microRNAs in fecal samples could be critical to understand physiological and pathological conditions. They could even be used as a potential marker for a new therapeutic strategy. However, the expression profiles and functions of the intestinal microRNAs present in the fecal samples of patients with HCC remains unclear.

Due to the instability of microRNAs, their low expression level and minor differences in sequence, highly sophisticated analytical methods are required. Quantitative real-time PCR (qRT-PCR) plays an important role in studying the biological functions of microRNA with strong specificity and high sensitivity (Daud and Scott, 2008).With the development of qRT-PCR, the selection of reference genes is crucial for the accuracy of the relative quantification of gene expressions (Liu et al., 2018; Zhou et al., 2018). However, only a few studies have been published that systematically evaluated the normalization targets in intestinal microRNAs qRT-PCR assays. Successfully screening for suitable reference genes first will provide reliable evidence for the quantitative analysis of intestinal microRNAs in fecal samples. Once the expression profile of intestinal microRNAs in HCC is confirmed by qRT-PCR, the functions of differentiated intestinal microRNAs could be investigated during the biological process.

This study aimed to find relatively stable reference genes and to clarify whether intestinal microRNAs from fecal samples play an important role in indicating HCC development. RNA was extracted from mouse fecal samples and microRNA expressions were detected by qRT-PCR. Four conventional housekeeping genes and 18 microRNAs were selected as candidate genes. Their expression stability was evaluated using GeNorm, NormFinder, and Bestkeeper software. These statistical analysis software programs were primarily used to identify appropriate reference genes in qRT-PCR experiments. We identified three genes (*mir-23a*, *GAPDH* and *let-7i*) which may be used as suitable reference genes for intestinal microRNAs. Next, we detected the expression levels of some intestinal microRNAs, which had already been observed in humans and mice, between the control and HCC group. Finally, we analyzed the mRNA targets of the differentiated intestinal microRNAs. Then GO terms and KEGG pathways were built using these databases. In this study, relatively suitable reference genes of intestinal microRNAs in fecal samples are first screened for, with a potential diagnosis and treatment for patients with HCC is then proposed.

## Materials and Methods

### Samples Preparation and the Ethics Approval

Professor Feng Hai from Harbin Medical University supplied fecal samples from five control and five HCC-suffering mice. The C57BL/6 mice (14th postnatal days) were injected DEN at a single intraperitoneal dose of 25 mg/kg and the animals were observed until 10 months. At 10 months of age, mice injected with DEN developed striking liver HCCs with tumors that could be clearly observed. The mice were allowed to defecate normally, and the first three fecal pellets of each animal were collected into an empty 1.5 ml tube with a sterile toothpick. A new toothpick was used for each mouse. The tubes were closed immediately and placed into liquid nitrogen, finally transferred into a −80°C refrigerator for storage. The methods were performed in accordance with the National Guidelines for Experimental Animal Welfare (the Ministry of Science and Technology, People’s Republic of China, 2006). All experimental protocols were pre-approved by the Experimental Animal Ethics Committee of Harbin Medical University, China.

### Total RNA Extraction

One ml of Trizol isolation reagent (Invitrogen, Carlsbad, CA) was added per 40–60 mg of frozen fecal samples and total RNAs was extracted according to the manufacturer’s protocol. The only difference from the usual RNA extraction protocol used in fecal samples was the need for repeated extraction using chloroform. The concentrations and purity of the total RNA extracted were measured by ultraviolet spectrophotometer NanoDro 2000 (Thermo Scientific, Waltham, USA). Purity requirements were considered to be met if the extracted RNA had an A260/A280 value between 1.80 and 2.00.

### Quantitative Real-Time PCR Assays

For reverse transcription, 1 μg of total RNA was reverse transcribed using a ReverTra Ace^®^ qPCR RT Kit (Toyobo, Osaka, Japan). The reverse transcription used a 20 μl reaction with random primers or gene-specific stem-loop primers, designed as described previously (Chen et al., 2005). All microRNA RT primer sequences are shown in **Table S1**. At the beginning of the experiment, the amplification efficiencies of the primers were first tested. According to the relative standard curve, the amplification efficiency of primers was about 93–116%. The cDNA was amplified by real-time PCR using SYBR^®^ Green Realtime PCR Master Mix (Toyobo, Osaka, Japan) on the ABI 7500 fast Real Time PCR system (Applied Biosystems, Carlsbad, CA, USA). The primer sequences of the primers (Invitrogen, Shanghai, China) are listed in **Table S1**. The quantitative RT-PCR reaction mixture was run in a 20 μl volume reaction. The mixture consisted of 10 μl SYBR Green PCR Master Mix, 1.0 μl of each specific primer, 2.0 μl cDNA template, and 6.0 μl RNase-free water. The reactions performed were as follows: 1 min at 95°C for pre-denaturation, 40 cycles of 15 s at 95°C for denaturation, 15 s at 60°C for annealing, 45 s at 72°C for extension.

### Data Preprocessing

Bestkeeper uses the cycle threshold (Ct) value directly from the qRT-PCR to analyze the stability of the gene, whereas the Ct value needs to be converted to a Q value before analysis by GeNormer and NormFinder. The formula for calculating the Q value is: *Q = 2^^-(Ct/sample-Ct/min)^*. The calculated results were then imported into Excel according to the rules of GeNormer and NormFinder as they only identify a specific format (Vandesompele et al., 2002, Andersen et al., 2004).

### MicroRNA Target Prediction and Functional Classification

The miRTarBase database (http://miRTarBase.mbc.nctu.edu.tw/) is a commonly used database for microRNA-target interactions. Using miRTarBase, all the validated mRNA targets of the differentially expressed intestinal microRNAs of control and HCC mice were listed. To create more accurate and reliable predicted results, only target genes validated by at least one experimental method were chosen. The DAVID Bioinformatic Resources database (https://david.ncifcrf.gov/) was then used to analyze the mRNA targets for enrichment of GO terms and KEGG pathways. A P value of <0.05 was used as the significance cut-off.

### Statistical Analysis

All measurement data were presented as mean ± SEM and analyzed by GraphPad Prism 5. Differences between groups were analyzed by Student’s t-test, where P< 0.05 was considered as indicative of a statistically significant difference.

## Results

### A Schematic Diagram for the Experimental Process

**Figure 1:**
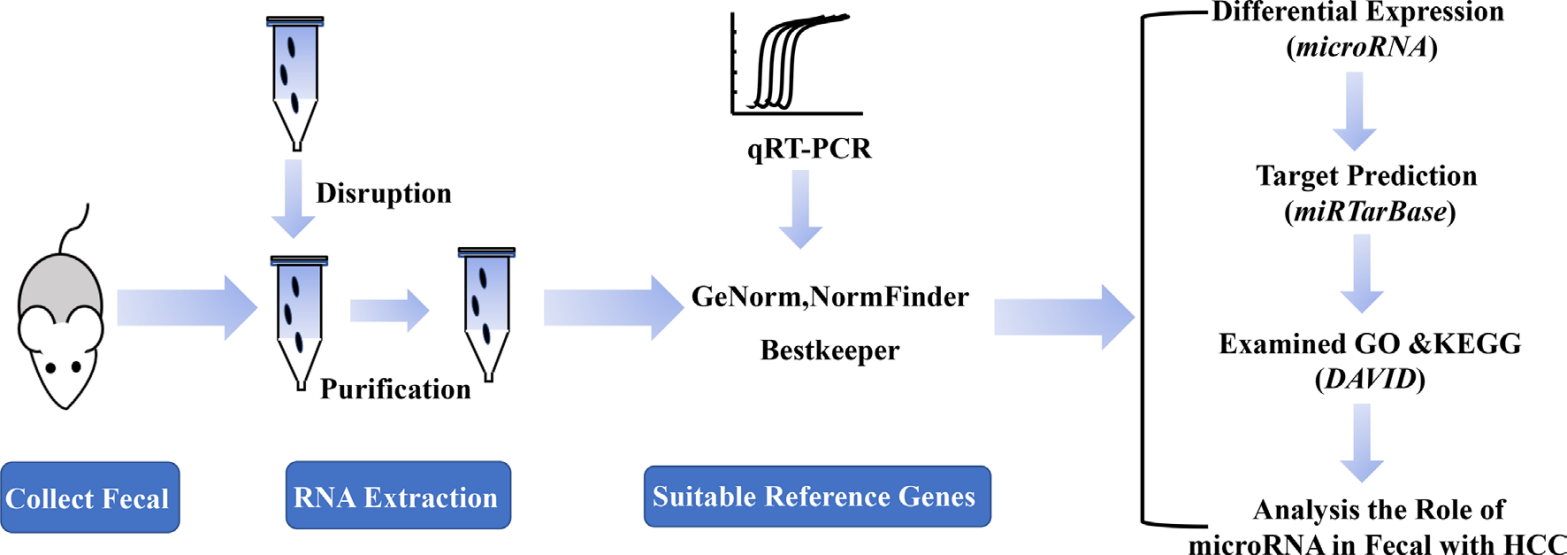
A schematic diagram of the experimental process.

In order to identify the suitable reference genes for intestinal microRNAs in fecal samples and to systematically analyze intestinal microRNAs expression in HCC, a schematic flow diagram of sample processing and microRNA analysis was first designed (**Figure 1**). Due to the novelty and scarcity of data in this area of study, 17 intestinal microRNAs, commonly expressed both in human and mouse fecal samples, were selected as candidate genes (Liu et al., 2016). Generally, *GAPDH*, *U6 snRNA*, *16S rRNA* and *5S rRNA* were also selected as they are usually used as reference genes.

### Comparison of Ct Values Between Candidate Reference Genes

**Figure 2:**
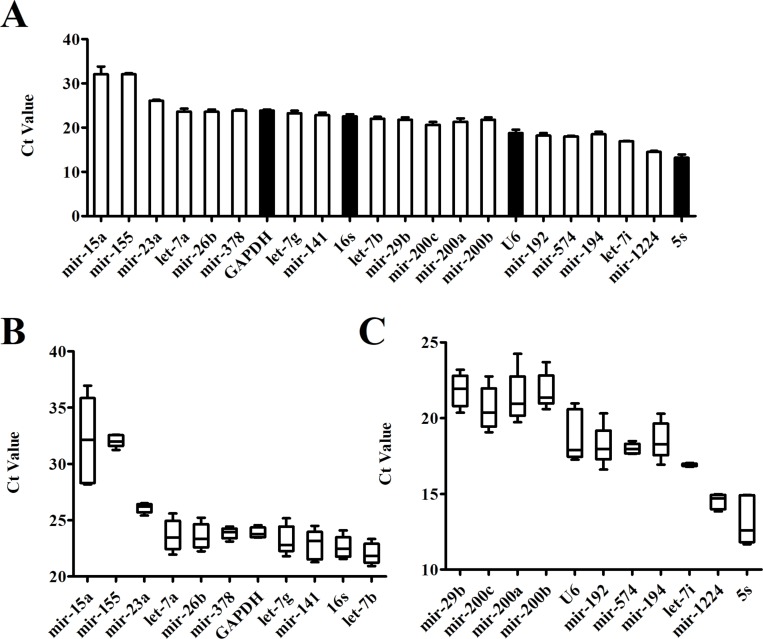
Expression level of candidate genes by real-time PCR. **(A)** Cycle threshold(Ct) values for the 22 candidate reference genes, n = 5. **(B**, **C)** Distribution of Ct values of candidate reference genes. Box representative the range of the Ct value distribution.

Even in different tissues or different growth stages of the same organ, there may be differences in the expression of the same gene. The Ct value of the gene is inversely proportional to the amount of target nucleic acid present. The lower the Ct value, the higher the amount of the gene present. According to its Ct value from real-time PCR, we analyzed the expression of each candidate reference gene (**Figure 2A**). The Ct values of 22 candidate reference genes ranged between 11 and 37. Large differences and a wide distribution range were found between the expressions of the candidate genes. The traditional reference genes *5S rRNA* had the highest expression level, where the Ct value was between 11.67-14.94; the expressions of *U6 snRNA*, *mir-192*, *mir-574*, *mir-194*, *let-7i*, and *mir-1224* were relatively high; *Mir-15a* and *mir-155* showed a larger Ct value with low expressions. Additionally, the box plots illustrated that the expression range of each candidate reference gene varied significantly (**Figures 2B, C**). The Ct value of *mir-155*, *mir-23a*, *mir-378*, *GAPDH*, *mir-574*, *let-7i* and *mir-1224* had fluctuating ranges that were comparatively small with a more concentrated distribution. Preliminary Ct values showed that the expression of *mir-23a*, *GAPDH*, *mir-574*, *let-7i*, and *mir-1224* was higher and had few differences between the samples.

### Expression Stability of Candidate Reference Genes

**Figure 3:**
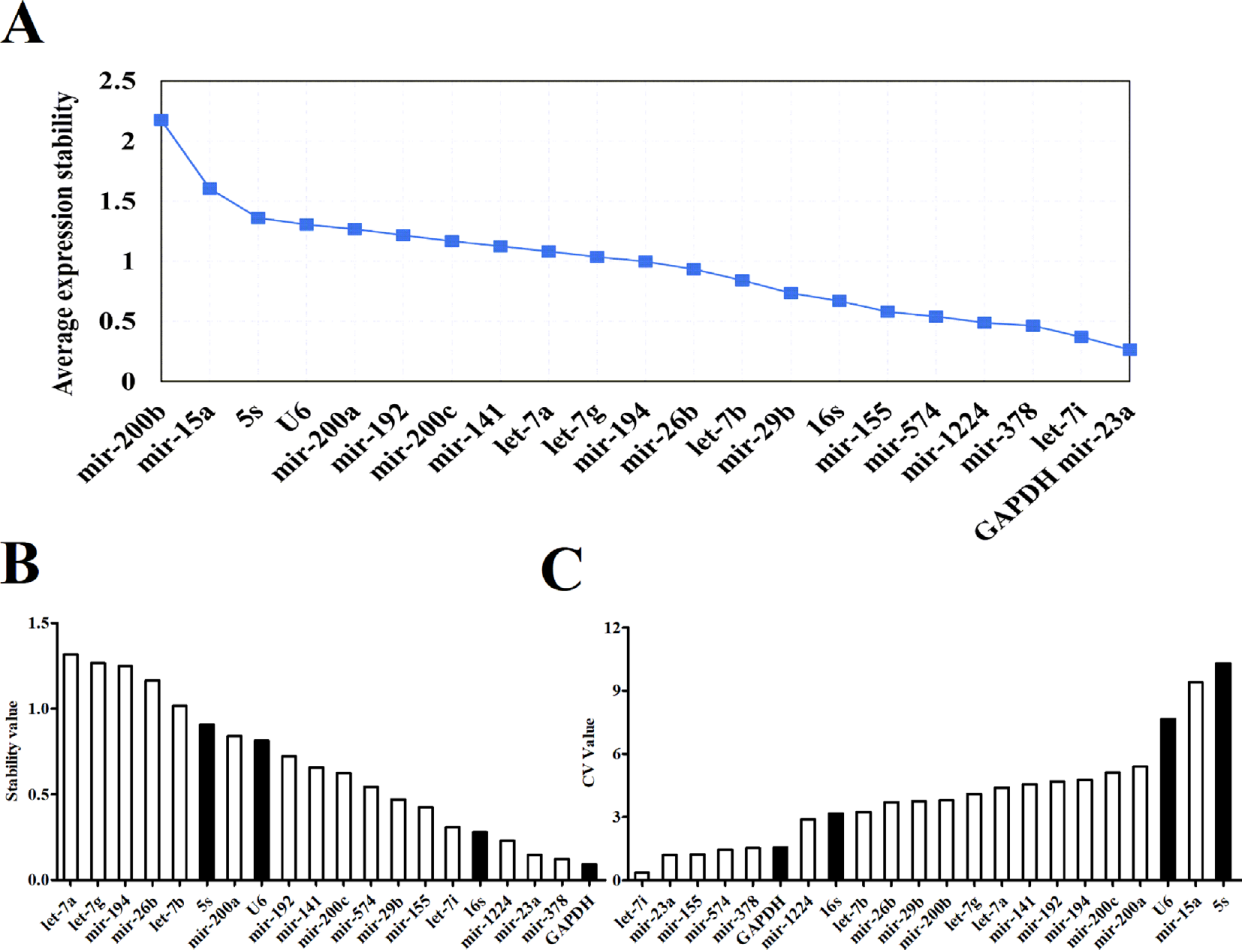
The expression stabilities of candidate reference genes analyzed by software. **(A)** Average expression M values analysis of candidate reference genes by geNorm software. **(B)** Average expression stability values analysis of candidate reference genes by NormFinder software. **(C)** Average expression CV values analysis of candidate reference genes by Bestkeeper software.

Three software packages (GeNorm, NormFinder, and Bestkeeper) were used for analyzing the average expression stabilities of the candidate reference genes. GeNorm and NormFinder software was specifically used for selecting reference genes. Before the analysis, it was necessary to convert the qRT-PCR result (Ct value) to a Q value. GeNorm software was written by Vandesompele in 2002 for screening reference genes where two or more reference genes can be chosen (Vandesompele et al., 2002). For the screening and selection of reference genes, an M value is usually displayed according to the output file of the GeNorm program. The default critical M value of the software is 1.5, where an M value of less than 1.5 indicates that the gene expression is relatively stable. The smaller the M value, the more stable the gene is. Through GeNorm software, we analyzed the M values of 22 candidate reference genes (**Figure 3A**). The data showed that the M value of *mir-200b* was the largest, whereas *GAPDH* and *mir-23a* were the smallest, indicating that *GAPDH* and *mir-23a* were the most stable genes. However, the traditional reference genes *U6 snRNA* and *5S rRNA* remained rather unstable. Another program, NormFinder, was written in 2004 by Claus, and was used to screen the stability of qualitative reference genes (Andersen et al., 2004). The calculation principle of this program is similar to that of GeNorm and the stability value used is also the M value. The results showed that *mir-23a*, *mir-378*, and *GAPDH* were relatively more constant (**Figure 3B**). *Mir-200b* and *mir-15a* were discarded in the results since they had M values that were too large. These results were similar to those obtained from the GeNorm software. The Bestkeeper software was also used to evaluate the stability of the candidate genes (Pfaffl et al., 2004). The Ct values of 22 candidate reference genes were calculated, along with the standard deviation (SD) and coefficient of variation (CV). Our results showed that higher gene stability is related to a smaller SD and CV. The data showed that *let-7i* had the lowest SD and CV values of 0.06 and 0.36, respectively, followed by *mir-23a*, *mir-574*, and *GAPDH* (**Figure 3C**, **Table S2**). The results from Bestkeeper were somewhat similar to those from GeNorm and NormFinder.

### Comprehensive Evaluation of Candidate Reference Genes in the Three Software Programs

When comparing the results of the three software programs, we found that despite the similar results obtained, there were still differences among them. A geometric mean method was thus used to evaluate the stability of each candidate reference gene. The candidate genes’ stabilities were ranked in each of the software programs and then the rankings were averaged across the three software programs (**Table 1**). The top three genes were *mir-23a*, *GAPDH* and *let-7i*. These three genes were found to be the most suitable reference genes for intestinal microRNAs. Considering that the expression and distribution of *let-7i* was the most concentrated and the highest, it was selected as a reference gene for the subsequent analysis. To further identify the stability of *let-7i* as a reference gene, a validated experiment was carried out in different animal models. We collected the fecal samples from some disease models whose incidence is higher, such as HCC, Type 2 diabetes (T2DM) and Myocardial Infarction (MI). The Ct value dispersion of the *let-7i* was detected by qRT-PCR. The result showed that there was no significant difference between different disease animal models (**Figure S1A**). Additionally, the boxplot also indicated that the distribution of *let-7i* was concentrated within groups (**Figure S1B**). These assays demonstrated that *let-7i* displayed higher stability which could be used as a reference gene for microRNA qRT-PCR in fecal samples.

**Table 1:** The overall ranking of expression stability for candidate reference genes.

Gene name	GeNorm	NormFinder	BestKeeper	Average	Comprehensive rankings
mir-23a	**1**	3	2	2	1
GAPDH	1	**1**	6	2.667	2
let-7i	3	6	**1**	3.333	3
mir-378	4	2	5	3.667	4
mir-1224	5	4	7	5.333	5
mir-155	7	7	3	5.667	6
mir-574	6	9	4	6.333	7
16S rRNA	8	5	8	7	8
mir-29b	9	8	11	9.333	9
let-7b	10	16	9	11.667	10
mir-26b	11	17	10	12.667	11
mir-141	15	11	15	13.667	12
mir-200c	16	10	18	14.667	13
mir-192	17	12	16	15	14
let-7g	13	19	13	15	15
mir-194	12	18	17	15.667	16
let-7a	14	20	14	16	17
mir-200a	18	14	19	17	18
U6 snRNA	19	13	20	17.333	19
mir-200b	22	22	12	18.667	20
5s rRNA	20	15	22	19	21
mir-15a	21	21	21	21	22

### Altered Intestinal MicroRNAs Expression Profiles in HCC Mice

**Figure 4:**
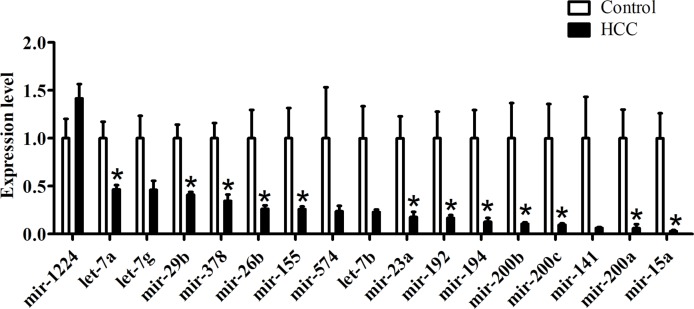
qRT-PCR validations of intestinal microRNA expression within HCC mice, n = 5, *P < 0.05.

In order to explore the functional intestinal microRNAs in HCC, we detected the expression levels of microRNAs which had been observed both in humans and mice. According to the comprehensive evaluation of candidate reference genes in the three software programs and their expression levels (Ct value), we chose *let-7i* as an internal reference gene. We demonstrated the presence of 11 miRNAs that displayed a decrease (*miR-155*, *miR-378*, *mir-23a*, *mir-26b*, *mir-29b*, *mir-194*, *mir-192*, *mir-15a*, *let-7a*, *mir-200a*, and *mir-200b*), in the HCC group compared with the control group, while *mir-200c*, *mir-1224*, *mir-574*, *mir-141*, *let-7b*, and *let-7g* showed no significant differences between the groups (**Figure 4**).

### Enrichment of Predicted mRNA Targets for Differentiated Intestinal MicroRNAs

**Figure 5:**
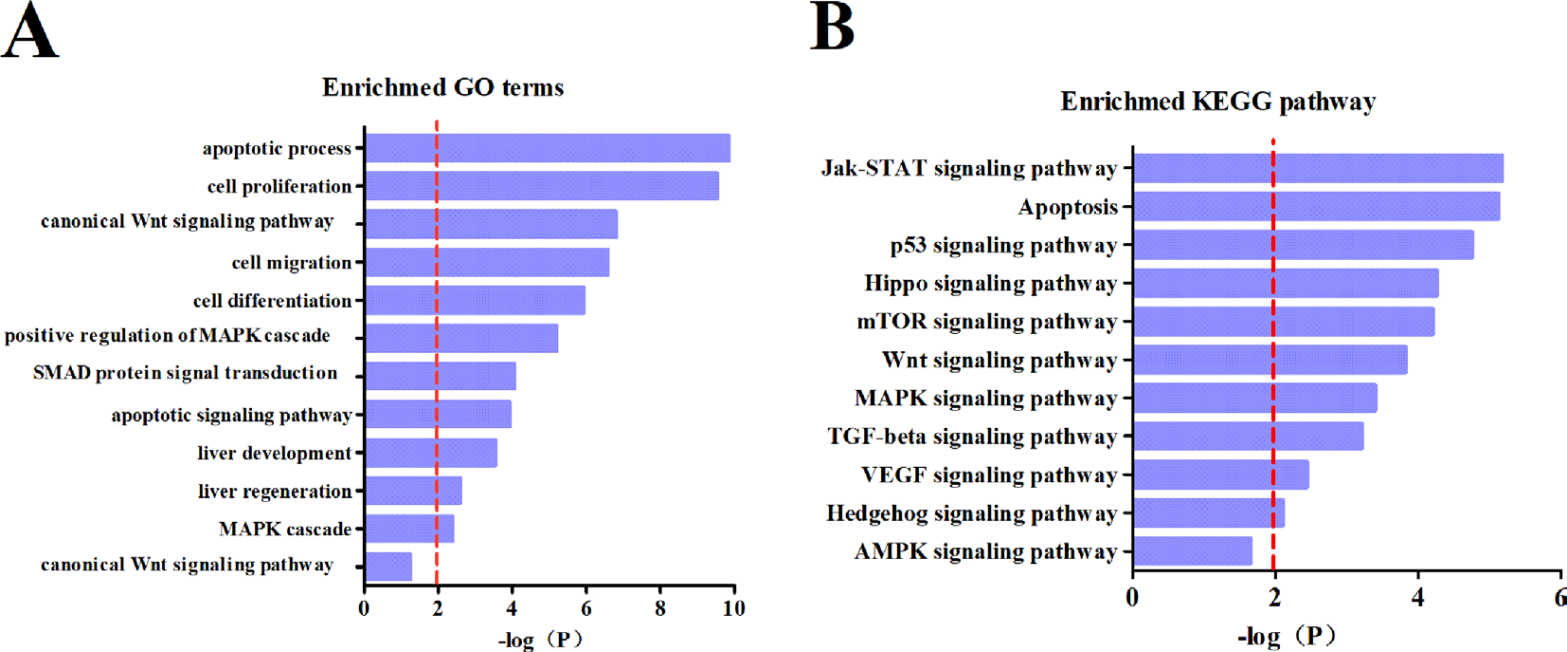
Subpathway enrichment analysis of microRNA target. **(A)** Enrichment of GO terms. **(B)** Enrichment of KEGG pathways.

To clarify downstream target gene networks for the intestinal microRNAs with HCC, we first listed all the potential mRNA targets of the differentiated intestinal microRNAs. These were predicted using miRTarBase. In order to improve the accuracy of the predicted mRNA targets, we only chose targets with strong evidence which had been supported by the reporter assay, western blotting or qPCR. The GO terms and KEGG pathways were then examined using the DAVID database. The results obtained from GO terms showed that the major processes in the pathology of HCC includes apoptosis, cell proliferation and the Wnt signaling pathway (**Figure 5A**). The KEGG pathway analysis explained that mRNA targets were mainly concentrated in the Jak-STAT pathway, apoptosis, and the p53 signaling pathway (**Figure 5B**). These results suggested that the differential intestinal microRNAs are likely to indicate the occurrence of HCC through predicted targets and pathways.

## Discussion

MicroRNAs are expected to become potential biomarkers for cancer diagnosis and new targets for disease treatments as aberrant expressions of microRNAs may be related to specific diseases. In recent years, quantitative RT-PCR assays have become the main method of studying the relative expression of microRNA. However, the quality of the results may be affected by some non-specific variables, such as the quality of RNA, efficiency of PCR amplification and the selection of reference genes (Yang et al., 2017). Appropriate normalization is a key part of quantitative gene expression analysis which is often ignored. The reference gene is also known as a housekeeping gene and is usually used to correct experimental errors when testing the gene expression levels. The ideal internal reference gene needs to satisfy the following conditions: the gene’s expression should be stable in different tissues, experimental conditions and growth stages (Lv et al., 2017, Molina et al., 2018). By using stable reference genes, the data in quantitative RT-PCR assays can be standardized. However, no reference genes have been found to be stable under all experimental conditions. Therefore, when studying specific gene expressions, it is necessary to select the most stable gene as the reference gene to correct the data and to ensure the accuracy of the results.

Interestingly, a new study suggests that fecal microRNAs could be identified as potential markers for intestinal malignancy (Ahmed et al., 2009). These fecal microRNAs exist in the gut lumen and feces and are called intestinal microRNAs. Some specific microRNAs that have been found in HCC cells and tissues may participate in tumor development, or could be a notable biological feature (Nelson and Weiss, 2008). The expression of microRNA in feces is therefore likely to become a new diagnostic marker in HCC. To achieve this potential, relatively accurate expression profiles of intestinal microRNA in HCC is a critical requirement. Because the reference gene of intestinal microRNA has not been identified yet, our first process step was to seek out a stable normalizer for the subsequent microRNA qRT-PCR experiments. Beginning with screening potentially stable reference genes, four traditional reference genes (*5S rRNA*, *U6 snRNA*, *GAPDH and 16S rRNA*) and 22 intestinal microRNAs co-expressed in humans and mice were used as candidate genes. Considering that normal samples are necessary in all experiments and conditions, we initiated our studies using control mice. First of all, the dispersion of the candidate genes’ Ct values was detected by qRT- PCR. There was a particularly large range of Ct values from 11 to 37 (**Figure 2A**). The boxplot indicated that Ct value distribution of *let-7i* was the most concentrated whereas *mir-23a*, *mir-378* and *GAPDH* had slightly dispersed Ct values (**Figures 2B, C**). Preliminarily assays demonstrated that these four genes with little dispersion also displayed higher stability.

However, the Ct value distribution map could only roughly assess the stability of the candidate reference genes. In order to determine the stability of a reference gene more accurately, it is necessary to further evaluate it using reference gene analysis software. There are a number of software programs available for analyzing reference genes, including GeNorm, NormFinder and Bestkeeper. They are three different statistical models that are widely used to select the most appropriate reference gene (Shen et al., 2011). Both GeNorm and NormFinder evaluates the stability of the gene by measuring the M value. When the maximum expression of the gene’s stability is achieved, it will be accompanied by a low M value. Bestkeeper is a comprehensive evaluation software program which depends on the SD and CV. The principle of determination is that the smaller the SD and CV is, the better the stability of the reference gene is. When SD is greater than one, it directly indicates that the expression of the reference gene is unstable. In our study, the stability of reference genes was analyzed by all three of these software programs. According to the qRT-PCR results, NormFinder showed that *GAPDH* was the most stable reference gene with the smallest M value (**Figure 3B**). This result was consistent with GeNorm software, with only one difference, GeNorm had the two best candidate reference genes and the other one was *mir-23a* (**Figure 3A**). This is in accordance with previous studies where *mir-23a* was considered to be a novel microRNA normalizer in profiling studies of cervical tissues (Shen et al., 2011). Nevertheless, the results of Bestkeeper were slightly different from those of GeNorm and NormFinder. The results showed that *let-7i* was one of the best reference genes with the smallest SD and CV (**Figure 3C**, **Table S1**), and was thus considered the most stable gene by the Bestkeeper software. Since different software programs have different algorithms, some differences are expected between the results obtained from each program. Therefore, to obtain relatively stable reference genes, we comprehensively analyzed the results from the three software programs. As presented in **Table 1**, it can be seen that the stabilities of the expression levels of the 22 candidate reference genes from the highest to the lowest were *mir-23a* > *GAPDH* > *let-7i* > *mir-378* > *mir-1224* > *mir-155* > *mir-574* > *16S Rrna* > *mir-29b* > *let-7b* > *mir-26b* > *mir-141* *mir-200c* > *mir-192* > *let-7g* > *mir-194* > *let-7a* > *mir-200a* > *U6 snRNA* > *mir-200b* > *5s Rrna* > *mir-15a*. Commonly used conventional reference genes in our microRNA qRT-PCR assays, like *5S rRNA* and *U6 snRNA* were particularly unstable. This phenomenon indicates that traditional reference genes are not applicable to all samples. Therefore, gene stability should always be evaluated and verified before use. Conclusively, *mir-23a*, *GAPDH* and *let-7i* are the optimal reference genes for intestinal microRNA in normal mouse feces. This information could provide recommendations for intestinal microRNA qRT-PCR studies with diseases statuses or other conditions.

MicroRNAs could modulate multiple target mRNA degradations or translations in the same pathway. Therefore, it has been proposed to identify miRNA regulatory profiles through predicted target genes and established miRNA-mRNA expression modules (Fu et al., 2012). In order to explore the possible mechanism of intestinal microRNAs regulating HCC, we constructed the intestinal miRNA-mRNA expression modules. Intestinal microRNA expression levels with HCC were first detected by quantitative RT-PCR. *Let-7i* was chosen as the reference gene since it showed a higher expression (Ct < 15) than *mir-23a* and *GAPDH* (**Figure 2A**). Results showed that there were differential intestinal microRNAs in the HCC group compared to the control group (**Figure 4**). Unexpectedly, *mir-23a* displayed a significant change in the HCC group, even though it has never been reported that *mir-23a* is a novel microRNA normalizer for relative quantification in human uterine cervical tissues. Indeed, this also clarified that the expression of reference genes is not always stable, and an appropriate reference gene under one experimental condition may not be suitable for all experimental conditions.

To better understand the biological functions of dysregulation intestinal microRNAs in HCC, a functional analysis of miRNA-mRNA was performed. Thus, an analysis of the differential microRNAs for GO terms and KEGG pathways were conducted. The targets of the intestinal microRNAs were significantly enriched in cell proliferation, migration and differentiation, which is closely related to the TGF-beta, Wnt and VEGF signaling pathway that is involved in tumorigenesis, metastasis and therapy (**Figure 5A**). *miR-29b* plays a protective role in cardiac remodeling by targeting the TGF-beta/Smad3 pathway (Zhang et al., 2014). mTOR is the key point of various important signaling pathways in cells, as well as being activated in a subset of HCCs (Fikret et al., 2004). The enriched KEGG pathway indicated that differential intestinal microRNA targets were likely to be involved in hepatocellular carcinogenesis through the mTOR pathway (**Figure 5B**). Previous research has clarified that *mir-155* enhances mTOR activity in the progression of breast carcinomas (Martin et al., 2014). It is evident that mRNA targets of differential intestinal microRNAs were significantly enriched in the apoptotic process and apoptosis pathway (**Figure 5**), where apoptosis was indicated to be involved in liver cancer development. Low *mir-15a* expression has shown negative regulation by targeting *Bcl-2* in human leukemia that is directly or indirectly involved in the biological process of apoptosis (Calin et al., 2008). The above evidence supports that intestinal miRNA could be a potential oncogene and antioncogene that is involved in HCC development.

In conclusion, the top three genes that were comprehensively ranked (*mir-23a*, *GAPDH* and *let-7i*), provide recommendations for intestinal microRNA qRT-PCR studies with disease statuses or other conditions. Additionally, differential intestinal microRNAs in fecal samples were predicted to be a potential oncogene or antioncogene involved in HCC development and may provide a novel strategy for diagnosing HCC and other related diseases.

## Author Contributions

HW and YL conceptualized and designed the experiments in this study. CW, DL, and YY performed experimental procedures related to the study. MB, SM, and YJ proofread and discussed articles relating to this research. ZW provides great help for revise article. YB and BY provided fnancial support and reviewed experimental directions. All authors contributed to the generation of experimental data for this study.

## Conflict of Interest Statement

The authors declare that the research was conducted in the absence of any commercial or financial relationships that could be construed as a potential conflict of interest.

## Supplementary Material

Figure S1 The expression level of let-7i by real-time PCR. (A) Cycle threshold (Ct) values for let-7i, n=5. (B) Distribution of Ct values of let-7i. Box representative the range of the Ct value distribution.
