# Supplementary material for: Systematic Analysis of Intestinal MicroRNAs Expression in HCC: Identification of Suitable Reference Genes in Fecal Samples
Source: Front Genet. 2019 Aug 13;10:687. doi: 10.3389/fgene.2019.00687 (PMC6700738; doi:10.3389/fgene.2019.00687)
Supplement: Supplementary file 2 [file Table_1.docx]

| **Gene name** | **RT suquence** |  |  |
| --- | --- | --- | --- |
| mir-1224 | 5‘CTCAACTGGTGTCGTGGAGTC  GGCAATTCAGTTGCTCCACCTC3’ | Forward | 5’ACACTCCAGCTGGGGTGAG  GACTGGGGAGGT 5’ |
|  |  | Reverse | 5'-CTCAACTGGTGTCGTGGA-3' |
| mir-155 | 5‘CTCAACTGGTGTCGTGGAGTC  GGCAATTCAGTTGACCCCTATCA 5’ | Forward | 5’ACACTCCAGCTGGGTTAATG  CTAATTGTGAT 3’ |
|  |  | Reverse | 5'-CTCAACTGGTGTCGTGGA-3' |
| mir-194 | 5‘CTCAACTGGTGTCGTGGAGTC  GGCAATTCAGTTGTCCACATGG 3’ | Forward | 5’ACACTCCAGCTGGGTGTAAC  AGCAACTCCAT 3’ |
|  |  | Reverse | 5'-CTCAACTGGTGTCGTGGA-3' |
| mir-26b | 5‘CTCAACTGGTGTCGTGGAGTCG  GCAATTCAGTTGACCTATCC 3’ | Forward | 5’ACACTCCAGCTGGGTTCAAG  TAATTCAGGAT 3’ |
|  |  | Reverse | 5'-CTCAACTGGTGTCGTGGA-3' |
| mir-200a | 5‘CTCAACTGGTGTCGTGGAGTCG  GCAATTCAGTTGACATCGTT 3’ | Forward | 5’ACACTCCAGCTGGGTAACAC  TGTCTGGTAAC 3’ |
|  |  | Reverse | 5'-CTCAACTGGTGTCGTGGA-3' |
| let-7g | 5‘CTCAACTGGTGTCGTGGAGTCG  GCAATTCAGTTGAACTGTACA 3’ | Forward | 5’ACACTCCAGCTGGGTGAGG  TAGTAGTTTGTAC 3’ |
|  |  | Reverse | 5'-CTCAACTGGTGTCGTGGA-3' |
| mir-192 | 5‘CTCAACTGGTGTCGTGGAGTCG  GCAATTCAGTTGGGCTGTCAA3’ | Forward | 5’ACACTCCAGCTGGGCTGACC  TATGAATTGAC 3’ |
|  |  | Reverse | 5'-CTCAACTGGTGTCGTGGA-3' |
| mir-141 | 5‘CTCAACTGGTGTCGTGGAGTCG  GCAATTCAGTTGCCATCTTTA 3’ | Forward | 5’ACACTCCAGCTGGGTAACAC  TGTCTGGTAAAG 3’ |
|  |  | Reverse | 5'-CTCAACTGGTGTCGTGGA-3' |
| mir-574 | 5‘CTCAACTGGTGTCGTGGAGTCG  GCAATTCAGTTGACACACTCA 3’ | Forward | 5’ACACTCCAGCTGGGTGAGTG  TGTGTGTGTGA 3’ |
|  |  | Reverse | 5'-CTCAACTGGTGTCGTGGA-3' |
| let-7a | 5‘CTCAACTGGTGTCGTGGAGTCGG  CAATTCAGTTGAACTATACA 3’ | Forward | 5’ACACTCCAGCTGGGTGAGGT  AGTAGGTTGTAT 3’ |
|  |  | Reverse | 5'-CTCAACTGGTGTCGTGGA-3' |
| let-7b | 5‘CTCAACTGGTGTCGTGGAGTCGG  CAATTCAGTTGAACCACACA 3’ | Forward | 5’ACACTCCAGCTGGGTGAGGT  AGTAGGTTGTGT 3’ |
|  |  | Reverse | 5'-CTCAACTGGTGTCGTGGA-3' |
| mir-29b | 5‘CTCAACTGGTGTCGTGGAGTCGG  CAATTCAGTTGAACACTGATT 3’ | Forward | 5’ACACTCCAGCTGGGTAGCAC  CATTTGAAATCAG 3’ |
|  |  | Reverse | 5'-CTCAACTGGTGTCGTGGA-3' |
| mir-378 | 5‘CTCAACTGGTGTCGTGGAGTCGG  CAATTCAGTTGACACAGGAC 3 | Forward | 5’ACACTCCAGCTGGGCTCCTG  ACTCCAGGTCCT 3’ |
|  |  | Reverse | 5'-CTCAACTGGTGTCGTGGA-3' |
| mir-200b | 5‘CTCAACTGGTGTCGTGGAGTCGG  CAATTCAGTTGTCATCATTA 3’ | Forward | 5’ACACTCCAGCTGGGTAATAC  TGCCTGGTAATG 3’ |
|  |  | Reverse | 5'-CTCAACTGGTGTCGTGGA-3' |
| mir-23a | 5‘CTCAACTGGTGTCGTGGAGTCGG  CAATTCAGTTGGGAAATCC3’ | Forward | 5’ACACTCCAGCTGGGATCACA  TTGCCAGGGAT3’ |
|  |  | Reverse | 5'-CTCAACTGGTGTCGTGGA-3' |
| mir-15a | 5‘CTCAACTGGTGTCGTGGAGTCGG  CAATTCAGTTGCACAAACC 3’ | Forward | 5’ACACTCCAGCTGGGTAGCAG  CACATAATGGT 3’ |
|  |  | Reverse | 5'-CTCAACTGGTGTCGTGGA-3' |
| mir-200c | 5‘CTCAACTGGTGTCGTGGAGTCGG  CAATTCAGTTGTCCATCATT 3’ | Forward | 5’ACACTCCAGCTGGGTAATAC  TGCCGGGTAATGA 3’ |
|  |  | Reverse | 5'-CTCAACTGGTGTCGTGGA-3' |

**Table. S1.** Primer sequences
