# Supplementary material for: Systematic Analysis of Intestinal MicroRNAs Expression in HCC: Identification of Suitable Reference Genes in Fecal Samples
Source: Front Genet. 2019 Aug 13;10:687. doi: 10.3389/fgene.2019.00687 (PMC6700738; doi:10.3389/fgene.2019.00687)
Supplement: Supplementary file 3 [file Table_2.docx]

| **Gene name** | **Min（Ct）** | **Max(Ct)** | **Std dev(Ct)** |
| --- | --- | --- | --- |
| let-7i | 16.79 | 17.04 | 0.06 |
| mir-574 | 17.64 | 18.49 | 0.26 |
| mir-23a | 25.42 | 26.51 | 0.31 |
| GAPDH | 23.46 | 24.57 | 0.37 |
| mir-378 | 23.11 | 24.44 | 0.37 |
| mir-155 | 31.26 | 32.59 | 0.4 |
| mir-1224 | 13.83 | 14.98 | 0.42 |
| let-7b | 20.93 | 23.34 | 0.71 |
| 16S rRNA | 21.56 | 24.09 | 0.72 |
| mir-29b | 20.35 | 23.19 | 0.82 |
| mir-200b | 20.6 | 23.69 | 0.83 |
| mir-192 | 16.6 | 20.31 | 0.86 |
| mir-26b | 22.23 | 25.23 | 0.88 |
| mir-194 | 16.93 | 20.29 | 0.89 |
| let-7g | 21.81 | 25.18 | 0.95 |
| mir-141 | 21.26 | 24.5 | 1.04 |
| let-7a | 21.95 | 25.6 | 1.04 |
| mir-200c | 19.07 | 22.76 | 1.06 |
| mir-200a | 19.73 | 24.25 | 1.16 |
| 5s rRNA | 11.68 | 14.94 | 1.36 |
| U6 snRNA | 17.25 | 20.97 | 1.44 |
| mir-15a | 28.18 | 36.96 | 3.02 |

**Table.S2.** The results of Bestkeeper analysis.
